# Reinforcing Cotton Recycled Fibers for the Production of High-Quality Textile Structures

**DOI:** 10.3390/polym17101392

**Published:** 2025-05-19

**Authors:** Tiago Azevedo, Ana Catarina Silva, Gonçalo Machado, Diego Chaves, Ana Isabel Ribeiro, Raul Fangueiro, Diana P. Ferreira

**Affiliations:** 1 Centre for Textile Science and Technology (2C2T), University of Minho, Campus de Azurém, 4800-058 Guimarães, Portugal; tiago.azevedo@2c2t.uminho.pt (T.A.); diego.chaves@2c2t.uminho.pt (D.C.); afr@2c2t.uminho.pt (A.I.R.); rfangueiro@det.uminho.pt (R.F.); diana.ferreira@det.uminho.pt (D.P.F.); 2 Polopiqué, Rua da Baiona, Vilarinho, 4795-784 Santo Tirso, Portugal; goncalo.machado@polopique.pt

**Keywords:** textile waste, recycled cotton fibers, cross-linking agents, polymers, mechanical properties, sustainability

## Abstract

The textile industry is under increasing pressure to adopt sustainable practices due to the significant environmental impacts associated with fiber production, including high energy consumption, water usage, and substantial greenhouse gas emissions. The recycling of textile waste, particularly cotton, is a promising solution that has the potential to reduce landfill waste and decrease the demand for virgin fibers. However, mechanically recycled cotton fibers frequently demonstrate diminished mechanical properties compared to virgin fibers, which limits their potential for high-quality textile applications. This study explores the use of cross-linking agents (citric acid (CA) and sodium hypophosphite (SHP)), polymers (polyethylene glycol (PEG), chitosan (CH), carboxymethyl cellulose (CMC) and starch (ST)), and silicas (anionic (SA) and cationic (SC)) to enhance the mechanical properties of recycled cotton fibers. The treatments were then subjected to a hierarchical ranking, with the effectiveness of each treatment determined by its impact on enhancing fiber tenacity. The findings of this research indicate that the most effective treatment was starck (ST_50), which resulted in an enhancement of tenacity from 14.63 cN/tex to 15.34 cN/tex (+4.9%), closely followed by CA-SHP_110/110, which also reached 15.34 cN/tex (+4.6%). Other notable improvements were observed with CMC_50 (15.23 cN/tex), PEG_50 (14.91 cN/tex), and CA_50 (14.89 cN/tex), all in comparison to the control. In terms of yarn quality, the CA-SHP_110/110 treatment yielded the most substantial reductions in yarn irregularities, including thin places, thick places, and *neps* with decreases of 36%, 10%, and 7%, respectively. Furthermore, CA_50 exhibited moderate enhancements in yarn regularity, thin places (−12%), thick places (−6.1%), and *neps* (−8.9%). The results of this study demonstrate that combining CA with SHP, particularly when preceded by the heating of the solution before the addition of the fibers, results in a substantial enhancement of the structural integrity, strength, and overall quality of recycled cotton fibers. This approach offers a viable pathway for the improvement of the performance of recycled cotton, thereby facilitating its wider utilization in high-quality textile products.

## 1. Introduction

Cotton is the most prevalent natural fiber in the textile and clothing industries. Its strength and durability are attributed by cellulose, a polysaccharide of glucose units linked by β-1,4-glycosidic bonds, which form crystalline microfibrils. The chemical structure of cellulose, which is rich in hydroxyl groups (-OH), is responsible for cotton’s ability to absorb water and react with various chemical treatments, including cross-linking agents and polymers [1]. In 2024, its global production was estimated at 24.8 million tons, thereby establishing fiber as a staple resource in the textile industry [2,3]. Cotton is cultivated primarily in arid regions where the cultivation of other crops is challenging, and it is produced on a large scale in several key countries. The countries that produce the greatest quantity of cotton are China (24.2%), India (23%), Brazil (12.8%), the United States (10.6%), Pakistan (5.9%), Australia (4.4%), Turkey (2.8%), and Uzbekistan (2.5%) [3]. However, cotton fibers require substantial resources for cultivation, transportation, and processing, further amplifying their environmental footprint. This is evident in the extensive consumption of energy, water, and chemicals, which leads to the generation of significant amounts of wastewater [4,5]. In response to these environmental pressures, the textile industry is increasingly transitioning towards more sustainable practices [6]. On a global scale, the fashion industry is estimated to generate approximately 92 million tons of textile waste annually, with projections suggesting that this figure could rise to 134 million tons by 2030 if the current trend continues.

Textile waste recycling offers a promising approach to address environmental concerns, reducing the burden on landfill sites and diminishing the need for virgin fibers [2,7]. The most common mechanical recycling method to process textiles is shredding, a well-established process at the industrial scale for the textile waste recycling of cotton fibers. The process involves the mechanical breakdown of the textile structures into fibers, which are then reassembled into yarn and, subsequently, again into textiles [8,9]. However, the fibers obtained from this process present several challenges that can adversely impact the mechanical tearing procedure. The spinnability and strength of yarn are largely determined by the length and mechanical properties of the fibers. During shredding, fibers are shortened, often requiring the addition of virgin materials when producing new yarn to achieve the necessary strength and quality [10,11,12]. To address these issues, softeners are frequently applied to the textile waste prior to shredding. These softening agents enhance fiber flexibility and facilitate mechanical breakdown, enabling more efficient separation and processing of individual fibers. By reducing the stiffness and friction of the fibers, softeners help to limit fiber breakage and minimize mechanical resistance, streamlining the shredding process and supporting higher throughput [13]. While softeners initially enhance the flexibility and processability of textile fibers, they can also compromise the durability and functional properties of the final recycled fibers. Softeners coat the fiber surface, which can adversely impact subsequent chemical treatments that are crucial for reinforcing the strength, cohesion, and mechanical stability of the fibers [14].

Cellulose cross-linking is a key chemical process in the field of textiles. It enhances fiber stability through the formation of molecular bridges between cellulose chains, thereby significantly improving fiber durability, mechanical strength, and functional properties. As such, it constitutes an essential step in the treatment of textiles. Cross-linking agents can both self-polymerize to form three-dimensional polymer networks or directly bond with cellulose hydroxyl groups through covalent interactions [15]. Poly(carboxylic acids) have emerged as a highly promising alternative to replace toxic cross-linkers in cellulose-based fibers [16]. Among them, citric acid (CA) is widely recognized for its environmental friendliness and efficiency. The cross-linking process of CA with cellulose begins with its dehydration to form a cyclic anhydride, which then undergoes esterification with the hydroxyl groups of cellulose. This cross-linking reaction has been shown to impart a number of unique properties to cellulose, including flame retardancy, a high swelling ratio, improved wrinkle resistance, antifibrillation characteristics, and, in some conditions, improved tensile strength. Sodium hypophosphite (SHP) has been identified as a crucial catalyst in this process, accelerating the formation of cyclic anhydride intermediates and their subsequent reaction with cellulose hydroxyl groups. In combination with CA, SHP enhances CA decomposition under heat, promoting the reduction of unsaturated intermediates to methylsuccinic acid. This compound can then convert into a cyclic anhydride, which esterifies with cellulose hydroxyl groups to form stable cross-links. This mechanism not only strengthens cellulose fibers but also enhances their resistance to mechanical stress and environmental degradation [17,18]. In addition to CA, other poly (carboxylic acids) have been the subject of study in relation to their potential for cross-linking. Yang et al. demonstrated that maleic acid (MA), in the presence of SHP, effectively conferred wrinkle resistance to cotton fabrics. In their study, cotton fabric samples were treated with 6.0% MA and cured at temperatures ranging from 130 to 180 °C for 2 min. The cross-linking process occurs through the interactions between the H–P–H bonds in SHP and the carbon–carbon double bond in MA esterified to cellulose, resulting in the formation of molecular bonds that enhance dimensional stability and reduce fabric wrinkling [19]. However, this study focused on cotton fabric and did not investigate the impact of MA–SHP systems on mechanically recycled cotton fibers, which exhibit different surface characteristics. Moreover, several studies have explored the synergies between cellulose and a variety of biopolymers, including starch (ST), chitosan (CH), and carboxymethyl cellulose (CMC). The objective of these studies was to introduce functional enhancements and improve the mechanical and physicochemical performance of cellulose-based materials [20]. These biopolymer–cellulose interactions offer a number of advantages, including enhanced tensile strength, thermal stability, moisture retention, and processing performance. For instance, ST has been demonstrated to considerably improve fiber adhesion and tensile strength by reinforcing hydrogen bonding within the cellulose matrix [20]. In addition, the cross-linking of this material with citric acid has been demonstrated to enhance the mechanical integrity and water resistance of composites derived from recycled cellulose sources, such as paper pulp. This finding suggests that the material has potential for use in eco-friendly applications [21]. CH, a biopolymer that is renowned for its intrinsic antimicrobial properties, has been shown to enhance fiber durability and to confer bioactive functionalities. These properties are particularly advantageous in medical and hygiene-related textile applications [16]. CMC, due to its anionic nature and high hydrophilicity, contributes to better fiber dispersion, reducing agglomeration and leading to more homogeneous fiber structures. Furthermore, the water retention capacity of cellulose is augmented, a property that is particularly beneficial in moisture-sensitive applications, including biomedical dressings and absorbent materials [18,20]. Beyond these conventional biopolymers, recent studies have proposed the use of more structurally complex agents, such as carboxylated polyaldehyde sucrose (CPAS). This biopolymer has demonstrated potential for enhancing water uptake and mechanical properties in cellulose substrates, while maintaining biodegradability and biocompatibility—qualities that are highly desirable in functional textile applications. Nevertheless, these methodologies have been restricted thus far to model cellulose systems, with no assessment conducted in the context of mechanically recycled cotton fibers, where fiber morphology and reactivity differ significantly [22].

The utilization of alternative polymers has also been demonstrated to enhance the quality of textile fibers and facilitate the spinning process. Lindstrom et al. conducted a study on the pre-treatment of both fibers and fabrics with polyethylene glycol (PEG) 4000, reporting enhanced fiber cohesion and diminished fiber loss during mechanical tearing. However, it is important to acknowledge a key limitation of the study: namely, its exclusive focus on virgin fibers and fabrics, without evaluating the behavior of recycled fibers subjected to similar treatment conditions [23]. The findings indicated that the application of PEG facilitated the rotor spinning of recycled fibers, highlighting its potential to improve the quality and efficiency of textile recycling.

Recent studies have investigated the use of physical reinforcement strategies to enhance the temporary mechanical strength of cotton yarns during processing, in addition to covalently bonded chemical treatments. Immich and Araújo reported a significant increase in tensile strength—up to 56%—in cotton yarns through the application of a poly (vinyl alcohol) (PVA) coating followed by cross-linking with glutaraldehyde [24].

In addition to biopolymers and poly (carboxylic acids), silicas have attracted increasing attention for their potential to reinforce cellulose-based fibers, particularly due to their tunable surface chemistry and charge-dependent interactions. As proposed by Aziz et al., an approach was developed involving the incorporation of silica directly into viscose fibers during the fiber formation stage. This method has been shown to result in a substantial reduction in the flammability of the resulting fibers, obviating the need for conventional flame-retardant chemical finishes, which are recognized to give rise to environmental and health concerns [25].

As the field of textile recycling progresses towards the production of higher-value, durable products, it is imperative that innovations in fiber treatment and processing are pursued. Current research efforts are thus focused on enhancing the mechanical properties of recycled fibers through a range of methods, including chemical treatment, blending with virgin fibers, and the refinement of recycling processes to better preserve fiber integrity [26,27]. Despite the numerous approaches, there is a significant gap in the literature regarding the applicability of these processes in improving the performance of mechanically recycled cotton fibers.

The present study addresses these limitations by means of a systematic evaluation of ten practical surface treatment strategies, including eco-friendly combinations such as citric acid and sodium hypophosphite, applied directly to recycled cotton fibers. In contrast to earlier research, which was predominantly focused on virgin fibers or fabrics, this investigation specifically focuses on mechanically recycled cotton fibers.

The novelty of the present research lies in its comprehensive comparison of scalable low-impact chemical treatments and their capacity to significantly enhance fiber tenacity, yarn uniformity, and overall mechanical performance. The objective of this study is to ascertain the most effective treatment for enhancing the quality of yarns produced from recycled cotton blends, thereby contributing to the advancement of sustainable practices in the textile industry.

## 2. Materials and Methods

### 2.1. Materials

Grey recycled cotton fibers, identified by the company as “Recover Aluminum Recycled Cotton 02903” (Lot: 501223), were kindly supplied by Polopiqué S.A. (Felgueiras, Portugal) and selected for the purposes of this study. The fibers were subjected to a series of chemical treatments with the objective of enhancing their mechanical performance when incorporated into recycled yarns. All the reagents were of analytical grade and were prepared according to the specific conditions required for each treatment, as detailed in Section 2.2. The following reagents were used: citric acid (CA), sodium hypophosphite (SHP), chitosan (CH), carboxymethylcellulose (CMC), and polyethylene glycol (PEG, molecular weight 10.000) sourced from Sigma-Aldrich (St. Louis, MO, USA), soluble potato starch (ST) obtained from PanReac AppliChem (Nürnberg, Germany), and anionic (TANAFINISH^TM^ A) and cationic (TANAFINISH^TM^ 40119) silicas kindly supplied by Tanatex Chemicals (Ede, The Netherlands). In order to ensure the reliability of the results of mechanical and quality tests, all the fabric samples were pre-conditioned at a temperature of (20 ± 2) °C and a relative humidity of (65 ± 2) % prior to being tested, in accordance with ASTM D1776/D1776M—Standard Practice for Conditioning and Testing Textiles [28].

### 2.2. Treatment of the Fibers

The various treatments applied to the fibers were conducted using the exhaustion technique, a widely employed method in textile processing, particularly for dyeing and finishing. This technique consists of immersing the fibers in a treatment bath where chemical agents are gradually absorbed by the material over time under controlled temperature and agitation, ensuring uniform penetration of chemicals into the fibers, thereby enhancing the final properties of the textile material.

Prior to the treatment, the fibers were subjected to rinsing with distilled water to eliminate any residual impurities, including those resulting from the utilization of previous softening agents commonly used in the mechanical recycling: 300 g of raw fiber was washed with distilled water and the procedure was repeated 3 times. The fibers were dried at 40 °C for 24 h before the treatments.

The treatments were conducted using a laboratory Ibelus beaker dyeing machine (Labelus, IL-720, Braga, Portugal) with a bath ratio of 1:10 (300 g of rinsed fiber and 3 L of the reagent solution). The procedure was repeated 5 times to achieve a total of 1.5 kg of treated fiber. The treatments that were applied included the following (Table 1):

(i) Exhaustion at 50 °C for 1 h with five agents (PEG_50, CH_50, CMC_50, ST_50, and CA_50);
(ii) Exhaustion at 110 °C for 1 h with CA and a combination of CA and sodium hypophosphite (SHP) (CA-SHP_110);
(iii) Pre-heating CA and SHP to 110 °C for 30 min, followed by the addition of recycled cotton fibers for further exhaustion at 110 °C for 1 h (CA-SHP_110/110);
(iv) Exhaustion at 50 °C for 15 min with anionic (SA_50) and cationic (SC_50) silicas.

The treatment temperatures were chosen based on the thermal sensitivity of the recycled cotton fibers and the specific reactivity of each chemical agent. Treatments carried out at 50 °C allowed for milder processing conditions, minimizing thermal degradation while isolating the effects of the polymers and silicas used. In contrast, treatments with citric acid (CA) and sodium hypophosphite (SHP) were carried out at 110 °C, as this temperature is necessary to activate esterification reactions and promote effective cross-linking with cellulose, as supported by both the literature and preliminary experiments. Overall, the temperature conditions were individually optimized for each treatment to ensure chemical efficacy while maintaining fiber integrity.

The reagent solutions were prepared prior to treatment as follows: PEG was dissolved at 1% *w*/*v* (1 g/100 mL), corresponding to approximately 1 mM; CH was prepared at 0.05% *w*/*v* by dissolving in 1% *v*/*v* acetic acid with continuous stirring overnight at room temperature (~22 °C), resulting in a final concentration of approximately 5 µM based on an estimated molecular weight of 100,000 g/mol; CMC was dissolved at 0.2% *w*/*v* (8 µM, an average molecular weight of 250,000 g/mol) by stirring until completely dissolved; ST was prepared at 1% *w*/*v* by stirring in distilled water until fully dissolved; and CA and SHP were dissolved in distilled water at their working concentrations; anionic and cationic silicas were each prepared at a concentration of 1.5% *w*/*v*, and the pH of both solutions was adjusted to the desired range of between 5.0 and 6.0 using dilute acetic acid or sodium hydroxide, as required. This concentration of anionic and cationic silicas was selected based on manufacturer recommendations for the functional finishing of cellulosic fibers and the temperature was slightly milder than reported for the treatment of cotton with silica nanoparticles [29]. All the solutions were freshly prepared prior to each treatment to ensure consistency and reagent stability.

Subsequently, the fibers were rinsed with distilled water, dried at 40 °C for 24 h, and subsequently employed in yarn production. Recycled fibers without any treatment were used as a control.

### 2.3. Yarn and Knitted Fabric Production

The process involved a series of stages through which the treated recycled cotton fibers were transformed into finished yarns. Each yarn comprised a 70:30 ratio of virgin cotton to recycled fiber. The selection of this ratio was informed by preliminary spinning trials and prior experience, which indicated that it offered an optimal balance between spinnability, yarn quality, and recycled content. The initial trials incorporated higher proportions of recycled fiber; however, these led to excessive fiber breakage and irregular yarn formation. This indicated that the 70:30 blend was the most viable and reproducible option for achieving consistent processing and ensuring product quality.

The mixture was processed through a carding machine (Rieter, C4, Winterthur, Switzerland) to produce a homogeneous web, which was then transformed into a sliver with a count of 0.110 Ne. Subsequently, the sliver was subjected to an initial pass through the draw frame (Trutzschler, TD7, Mönchengladbach, Germany). The objective of this process was to standardize, straighten, and even out its color by bending and stretching. A second pass through the draw frame was conducted, resulting in a silver with a linear density of 0.120 Ne. Subsequently, the sliver was transformed in a roving frame (Rieter, F36, Winterthur, Switzerland) with a twist of 1.48 (T/”) and a draft of 7.5, resulting in a linear density of 0.90 Ne. The roving was continuously spun into yarn on a compact ring-spun yarn (Rieter, K46, Winterthur, Switzerland), with a twist of 810 (T/m) and a draft of 28.60. The completed yarn was then waxed in a winding machine (Saurer Schlafhorst, Autoconer 6, Übach-Palenberg, Germany) and five bobbins of each sample of 400 g were produced. Knitted fabrics (jersey) were produced using the produced yarns in the equipment Sodemat 270 Faubrog Croncels 10,000 Troyes (SODEMAT, Troyes, France) with a gauge of 20.

### 2.4. Characterization of the Fibers

#### 2.4.1. Scanning Electron Microscopy (SEM)

Scanning electron microscopy (SEM) was used to analyze the surface morphology of the obtained fibers. Surface and morphologic analyses of the recycled cotton fibers were carried out using a scanning electron microscope FlexSEM 1000 II (Hitachi, Japan). The samples were fixed to the stub using a conductive double-sided adhesive carbon and sputter-coated for 30 s with Au in a Quorum MiniQS sputter coater (Laughton, United Kingdom). The SEM analysis parameters were high vacuum mode using secondary electron detector (Bruker Nano GmbH, Berlin, Germany), 5 kV acceleration voltage, and spot size 30 at magnifications higher than 10,000×.

#### 2.4.2. Fourier Transform Infrared Spectroscopy with Attenuated Total Reflectance (FTIR-ATR)

Fourier transform infrared spectroscopy (FTIR) coupled with an attenuated total reflectance (ATR) accessory was used to evaluate changes in the chemical composition of the treated fibers. The sample analysis was performed using the IRAffinity S1 equipment from Shimadzu (Kyoto, Japan), equipped with an ATR accessory. Spectra were collected over 45 scan cycles and in the spectral range 4000–400 cm^−1^, with a resolution of 4 cm^−1^ in transmittance mode. A bundle of fibers from each method performed was submitted for analysis. Three measurements were made on each sample.

#### 2.4.3. Thermogravimetric Analysis and Differential Thermogravimetric Analysis (TGA/DTG)

A thermogravimetric analysis was used to access changes in the content of cellulose. The TGA/DTG of the fibers were performed in a Hitachi STA7200RV Thermal Analysis System (Tokyo, Japan), in the range of 25–600 °C at 10 °C min^−1^. The N_2_ flux was 200 mL min^−1^ and the initial sample weighed between 6.64 and 8.83 mg in alumina pans. Data were plotted as weight percentage versus temperature, and the mass of dried residues was calculated. The maximum peaks of the thermal transformation events were identified by performing the derivative thermogravimetric analysis.

#### 2.4.4. X-Ray Diffraction (XRD)

The treated recycled cotton fibers that promote superior mechanical properties to the corresponding yarns and the control sample were analyzed by XRD using a Bruker AXS D8 Discovery diffractometer (Karlsruhe, Germany). The diffractometer operated using Cu-Kα as a radiation source (λ = 1.5406 Å), accelerating voltage at 40 kV, current at 40 mA, and 2θ angle ranging from 5 to 55°, with the scan step size of 0.05° and 3 s per step. The crystallinity index (*I_c_*) was calculated according to the peak height method reported by Segal et al., through the following Equation (1) [30]:

(1) $$Ic\;(\%)=\frac{\left(I_{002}-I_{am}\right)}{I_{002}}\times 100\%$$

where *I_002_* is the maximum intensity, typically found between 2θ = 21° and 2θ = 23°, for crystalline cellulose, and *I_am_* is the intensity of the peak at 2θ = 18° for amorphous cellulose. By measuring the intensities of these peaks and applying the formula to calculate the crystallinity index, it is possible to determine the crystallinity of the cellulose fraction into the fibers under study. The crystallinity index provides information about the degree of crystalline and amorphous regions present in the cellulose material.

### 2.5. Characterization of the Yarn

#### Yarn Properties

Mechanical characterization was carried out using an Uster TensoRapid IV (Uster, Switzerland), Uster Tester 5 (Uster, Switzerland), Uster Zweigle Twist Tester 5 (Uster, Switzerland), and Uster Zweigle Yarn Reel (Uster, Switzerland), that could perform the tests as explicit on the ISO 17202 standard [31]. A total of 150 repetitions were conducted for each treatment. The tensile strength, elongation at break, and tenacity (cN/Tex) were calculated.

### 2.6. Fabric Characterization

#### 2.6.1. Air Permeability

In conducting this test, the principles of the ISO 9237:1997 standard [32], entitled Air Permeability of Fabrics, were adhered to. The air permeability tester TexTest FX 3300 (Schwerzenbach, Switzerland) was utilized in this process. The tests were repeated for each sample ten times, and a 20 cm^2^ test area and 200 Pa pressure were used. This equipment allows forcing air through the material under evaluation, thereby determining the pressure differential between its two sides. The test allows the quantification of the flow rate of air that traverses each sample, thus facilitating the determination of the material’s air permeability.

#### 2.6.2. Alambeta Test

The Alambeta device (Sensora s.r.o, Na vybezku, Czech Republic) was utilized in the evaluation of thermal properties, with the objective of assessing the sensation of heat or cold. This sensation is of significance not only in the momentary experience of a fabric, but also in the context of wearing any piece of clothing or footwear, and during the periodic contact of internal body parts with clothing. The Alambeta is a multifaceted instrument that facilitates the evaluation of both stationary thermal properties, such as resistance and conductivity, and the dynamic properties, such as thermal diffusivity and thermal absorption. The apparatus consists of a metal block with a constant temperature (32 °C), which differs from the sample temperature (20 °C). The Alambeta instrument evaluates a range of parameters, including thermal conductivity (λ), thermal resistance (R), thermal absorptivity (b), heat flow (qm), thickness (h), and thermal diffusivity (a), under a contact pressure of 200 Pa.

#### 2.6.3. Pilling Box

In accordance with the protocol outlined in ISO 12945-1:2020 [33], the knitted fabrics were meticulously prepared for the pilling box trial using an Trumeter PT 2 (Testrite, Ltd., West Yorkshire, United Kingdom). This involved a series of precise procedures, including cutting, sewing, and the subsequent insertion into cylindrical polyurethane tubes. The trial was conducted over a duration of four hours, after which the rating degree listed in Table 2 was meticulously measured.

## 3. Results and Discussion

### 3.1. Fiber Characterization

#### 3.1.1. SEM

**Figure 1:**
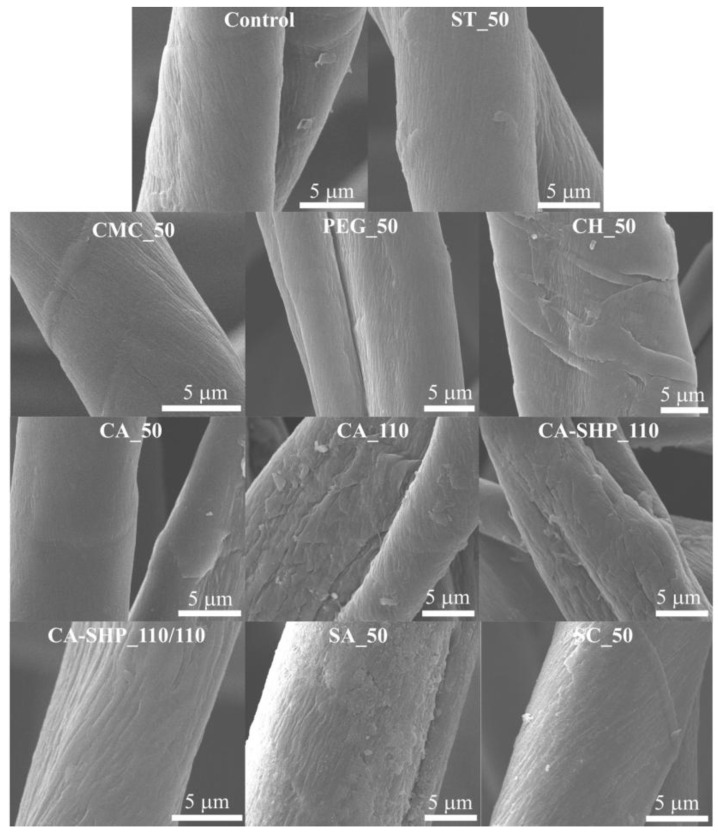
SEM images of the surface of recycled cotton fibers after different treatments.

SEM was employed to analyze the surface and morphology of the cotton fibers that had undergone chemical functionalization and the corresponding control sample (Figure 1). By analyzing SEM images at varying magnifications (Figure 1), it is possible to assess the distinctive attributes of the fibers, including the presence or absence of surface irregularities resembling scales or other irregular shapes along their length. The observed morphology through SEM imaging was consistent with that of cotton fibers, which exhibit a distinctive twisted ribbon-like structure [34,35]. In the SEM image of the control recycled cotton fiber, distinct grooves are visible on the fiber surface, accompanied by small areas of low roughness distributed in parallel segments, as well as surface irregularities resembling scales. These surface features are characteristic of cotton fibers and contribute to their well-known absorption and comfort properties. The SEM image of the washed recycled cotton fiber is similar to that of the control sample but lacks surface irregularities resembling scales observed in the untreated fiber (control). The SEM images of recycled cotton fibers treated with CA, CMC, and starch exhibit a morphology similar to that of the untreated cotton fiber, indicating that these treatments had a minimal impact on the fiber’s surface characteristics. In contrast, fibers subjected to treatments with CA_110, CA–SHP_110, and CA–SHP _110/110, as well as those treated with cationic and anionic silica, display more pronounced grooves on their surfaces. Moreover, the SEM images of the treated fibers with silica, more evident in SA_50, illustrated the formation of a discernible surface layer, which is indicative of its deposition.

#### 3.1.2. FTIR-ATR

**Figure 2:**
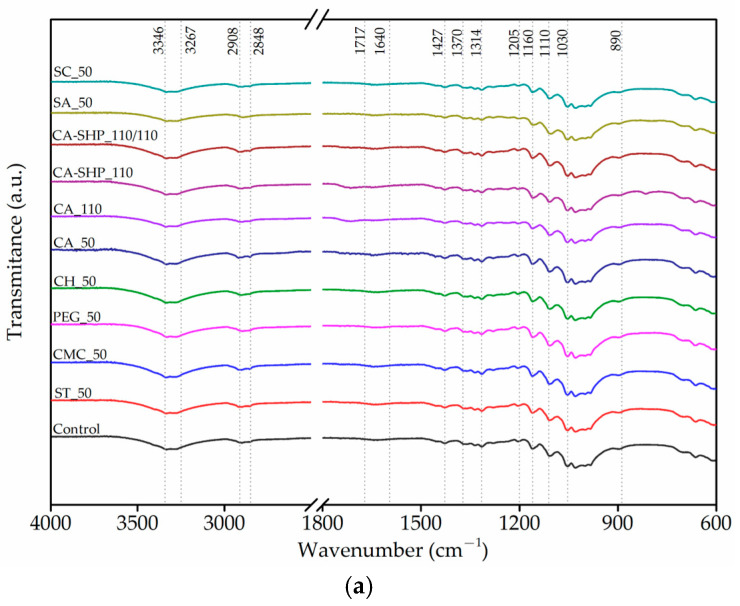

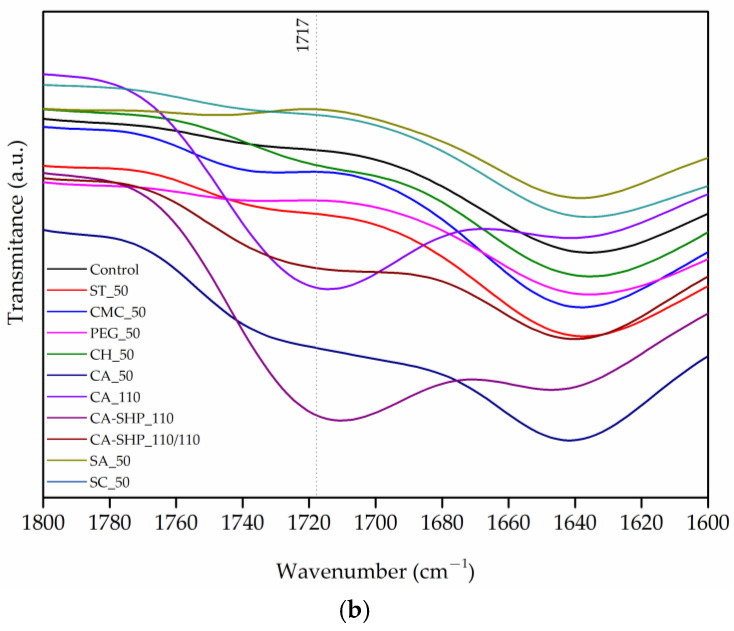
FTIR-ATR spectra of recycled cotton fibers untreated and with different treatments in the region 4000–400 cm^−1^ (**a**) and in the region 1800–1600 cm^−1^ (C=O stretching) (**b**).

The FTIR-ATR spectra of the recycled cotton fibers are shown in Figure 2. The analysis of the spectra reveals distinct bands corresponding to the components of cellulose, hemicellulose, and lignin, which are characteristic of lignocellulosic fibers [36]. No notable differences were discerned in the FTIR-ATR spectra of the treated and untreated fibers. The broad absorption band between 3346–3267 cm^−1^ is attributed to the O-H stretching vibration of the hydroxyl group, indicating the presence of absorbed water, free phenols, primary and secondary aliphatic alcohols found in cellulose, hemicellulose, and lignin [36,37]. The absorption bands at 2908 cm^−1^ and 2848 cm^−1^ correspond to the stretching vibrations of the aliphatic C-H group, specifically CH_2_, indicating the existence of cellulose and hemicellulose [36,37]. The absorption band at 1717 cm⁻¹ is indicative of the stretching vibration of the C=O bond in carbonyl groups, which can be attributed to the presence of acetyl groups, a typical constituent of hemicellulose [36,37]. The absence of the aforementioned peak at 1717 cm^−1^ is indicative of a relatively low hemicellulose content in recycled cotton fibers. However, in fibers treated with citric acid or with a combination of citric acid and sodium hypophosphite at 110 °C, the peak becomes evident, likely due to the formation of ester bonds between citric acid and the cellulose fibers. These treatments introduce additional carbonyl (C=O) groups through esterification, with sodium hypophosphite acting as a catalyst that enhances the bonding process, leading to an increase in the carbonyl-related absorption band at 1717 cm⁻¹. A peak around 1640 cm^−1^ is due to the adsorbed water molecules [38,39]. The absorption band at 1427 cm^−1^ is associated with the symmetric bending of CH_2_ of the cellulose [38]. The absorption bands at 1370 and 1314 cm^−1^ are relative to the asymmetric deformation vibration of the C-H group and the stretching vibration of C-O, respectively, due to the aromatic rings of cellulose [37,38,40]. The absorption bands at 1205, 1160, and 1110 cm^−1^ are due to OH in-plane bending and asymmetric stretching of the C-O-C of the β-glycosidic bond [37,38,40,41].

The absorption bands at 1032 cm^−1^ and 890 cm^−1^ correspond to the stretching vibration of C-O and O-H and the β-glycosidic bonds, respectively, associated with cellulose [33,34,36]. All the mentioned bands are summarized in Table 3.

#### 3.1.3. TGA/DTG

**Figure 3:**
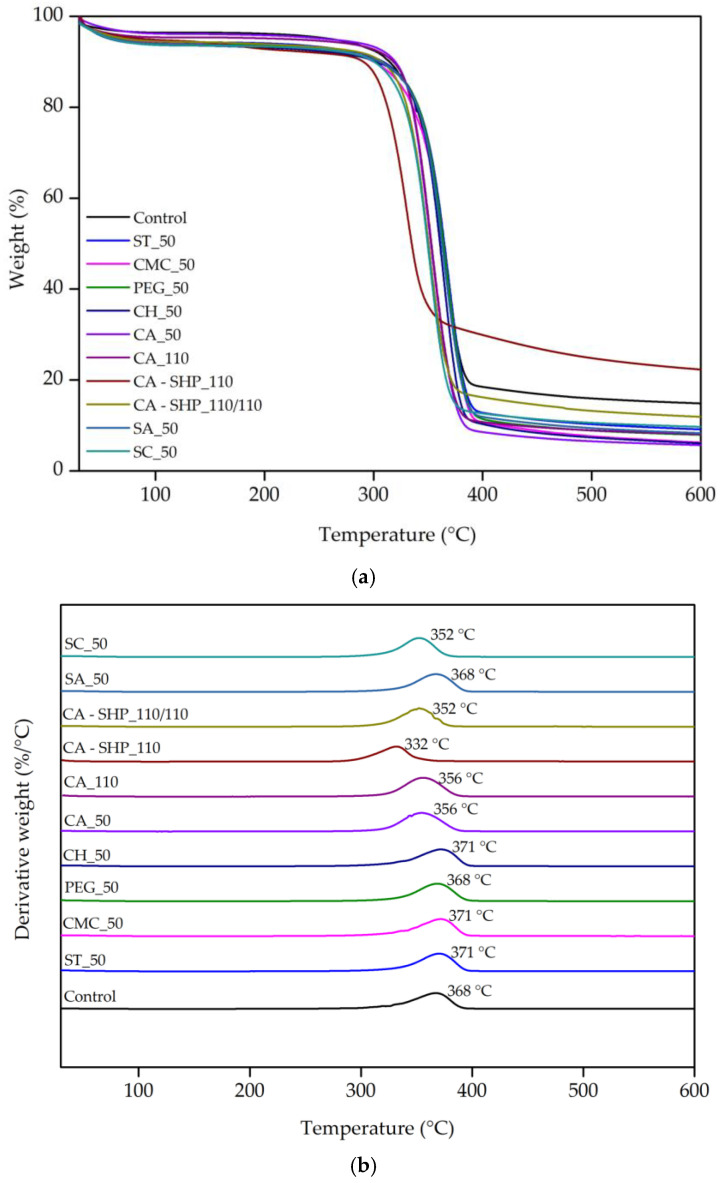
Thermogravimetric curves (**a**) and corresponding derivatives (**b**) of recycled cotton fibers untreated and treated samples under nitrogen atmosphere at a rate of 10 °C.min^−1^.

The TGA/DTG curves of the recycled cotton fibers display a comparable weight loss to those previously documented in the scientific literature for cotton fibers [34]. The TGA was carried out to evaluate the thermal stability and understand the degradation process of the cotton fibers with different treatments. The TGA together with the differential thermogravimetric analysis (DTG), which is the derivative of TGA, results are shown in Figure 3 and Table 4.

The thermal degradation process occurs in three primary stages, as seen in the mass loss curve (TGA), while the DTG analysis revealed a predominant degradation phase. The first stage (50–150 °C) corresponds to moisture evaporation and the removal of volatile compounds. A second phase, between 220 and 409 °C, encompasses the overlapping thermal degradation of hemicellulose and cellulose, which are closely related in their decomposition behavior and appear as a single peal in the DTG curve. The final stage (409 and 600 °C) is associated with the slow degradation of lignin, the oxidation of residual char, and the combustion of remaining carbonaceous materials. Although lignin degradation begins at lower temperatures (200 °C), its decomposition extends broadly up to 600 °C, contributing to the continued weight loss in this region [42]. The highest degradation rate for the majority of the samples is observed around 369 °C, indicating that this is the main thermal decomposition phase associated with cellulose breakdown (Table 4). However, the TGA analysis was unable to distinctly identify the peaks corresponding to hemicellulose and lignin degradation. This limitation may be attributed to the low amounts of these polymers in the cotton samples analyzed. Furthermore, it can be observed that certain treatments appear to exert an influence on the thermal stability of the fibers. To illustrate, the sample treated with CA-SHP_110 displays a peak at a lower temperature (332 °C) in the DTG analysis, indicating that this treatment may reduce the thermal stability of the recycled cotton fibers. The decrease in thermal degradation temperature to 332 °C observed in the CA-SHP_110 treatment is attributed to the simultaneous exposure of the cellulose fibers to increasing temperature and acidic conditions during the solution heating phase. This progressive heating in the presence of reactive acid species may enhance hydrolysis and chain scission of the cellulose backbone, resulting in reduced thermal stability, as evidenced by the FTIR spectrum. The analysis of the weight loss derivative (DTG) between 274 and 409 °C revealed an approximate weight loss of 80% which may be associated with the decomposition of cellulose. The majority of treated samples exhibited notable decomposition within the 350 to 370 °C range with specific peak temperatures indicated alongside each curve. These peaks indicate the main thermal degradation phase, which is likely due to the breakdown of cotton’s cellulose structure.

### 3.2. Yarn Characterization

The yarns displayed a linear density of 24 Ne with consistent reliability. A comprehensive examination of the treatments employed on recycled cotton fibers revealed notable discrepancies in their efficacy at enhancing mechanical properties, structural integrity, and overall quality (Table 5). The application of PEG_50 and CMC_50 resulted in a tenacity increase by 1.9% and 4.1%, respectively, in comparison to the control. However, both treatments resulted in a deterioration of yarn quality, with an increase in imperfections such as thin and thick places and *neps*. These defects resulted in a reduction in the uniformity and smoothness of the yarn, thereby highlighting a trade-off between enhanced strength and deteriorated structural consistency. In contrast, the treatment with chitosan yielded more complex results. While the treatment resulted in improvements in certain characteristics of the yarn, including a reduction in thick places and *neps*, it also introduced notable drawbacks. The chitosan treatment resulted in a reduction in yarn tenacity, a crucial measure of strength, and an increase in the occurrence of thin places. The combination of reduced strength and increased defects resulted in a deterioration of the yarn’s overall quality, rendering it less robust and uniform. The treatment with ST_50 resulted in the highest increase in tenacity, with a 4.9% improvement compared to the control (15.34 cN/tex versus 14.63 cN/tex), highlighting its ability to strengthen fibers. However, this enhancement in strength was accompanied by a significant increase in structural defects, including a 76% rise in thin places and a 21% increase in *neps*, indicating a trade-off between improved strength and reduced uniformity.

The application of citric acid (CA) resulted in a more balanced impact. At 50 °C, CA_50 treatment notably reduced the prevalence of thin places, thick places, and *neps*, corroborating previous findings on CA’s ability to improve the physical properties of natural fibers through cross-linking mechanisms [43]. At this temperature, the treatment resulted in an increase in yarn tenacity by 1.8% and a reduction in fiber irregularities, including a 12% decrease in thin places, a 6.1% decrease in thick places, and an 8.9% decrease in *neps*. This outcome represented an optimal balance between the enhancement of strength and the uniformity of the fibers, making it the most favorable single treatment. At 110 °C, the application of citric acid (CA_110) resulted in a further improvement in fiber uniformity, with a reduction in thin places by 38% and *neps* by 28%. However, this improvement was counterbalanced by a 6.4% reduction in tenacity, indicating a trade-off between structural consistency and strength. The lower temperature treatment at 50 °C achieved a superior equilibrium, although with a more modest increase in tenacity.

**Figure 4:**
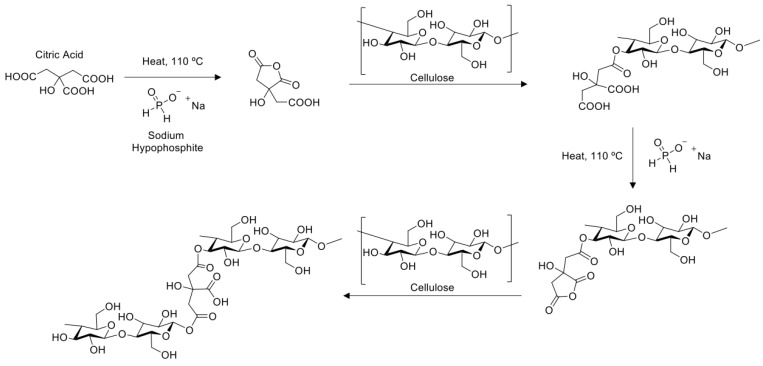
Mechanism of the occurrence of cross-linking between cellulose and citric acid with the addition of SHP by heating.

Wulandari et al. [44] propose that the combined process of CA-SHP involves the formation of ester bonds between the hydroxyl groups of cellulose and the carboxyl groups of citric acid (Figure 4). The reaction is initiated by the hydration of citric acid, to form a cyclic anhydride, which subsequently undergoes esterification with the hydroxyl groups of cellulose. In this context, the combined CA-SHP_110/110 treatment was conducted in two distinct ways, both at the same temperature (110 °C) and for the same duration (1 h), but differing in the timing of fiber addition. When the solution was preheated prior to the addition of the fibers, the cyclic anhydride formation was more efficient, as the acidic medium had already been partially activated. This enabled more effective cross-linking through robust ester bonds, resulting in a 4.6% increase in fiber tenacity and a reduction in yarn irregularities as follows: 36% fewer thin places, 10% fewer thick places, and 7% fewer *neps*. These results are particularly relevant when evaluated against industry benchmarks. According to the USTER^®^ statistics for Ne 24 ring-spun cotton yarns, the median tenacity (USP 50%) is approximately 14.0 cN/tex, with typical defect levels of 63 thin places, 344 thick places, and 344 *neps* per 1000 m. The performance of the CA-SHP_110/110-treated yarns was within or close to this percentile range, indicating that the treatment effectively restores the mechanical and structural properties of recycled cotton fibers to levels comparable to those typically observed in standard 70:30 virgin-to-recycled cotton yarns. This alignment with international quality benchmarks, such as the USTER^®^ statistics, reinforces the industrial relevance and scalability of the proposed treatment.

Conversely, when the fibers were added to the solution in the beginning of the process (CA-SHP_110), the formation of the cyclic anhydride was less effective, resulting in a weaker cross-linking process. Furthermore, the fibers experienced prolonged exposure to acidic and thermal stress, which may have induced partial hydrolysis or oxidative degradation of the cellulose chains. This is likely to have interfered with the cross-linking process, leading to a 7.7% reduction in tenacity and an increase in irregularities. The number of thin places increased by 49%, the number of thick places by 14%, and the number of *neps* by 5%. The role of sodium hypophosphite (SHP) in this process is crucial. SHP functions as a catalyst by enhancing the formation of cyclic anhydrides from citric acid through a process of dehydration. In addition to this, SHP may also function as a reducing agent, thereby stabilizing the reaction medium and preventing premature degradation of reactive intermediates. In optimal conditions, SHP accelerates esterification and improves the degree of substitution and cross-linking density, thereby enhancing fiber mechanical integrity. These observations underscore the necessity for the meticulous regulation of the reaction sequence and environment to optimize treatment efficacy while minimizing cellulose degradation.

The treatments with silica (both anionic and cationic) yielded results that were not as favorable as those observed with the CA and SHP treatments. While the silica treatments resulted in a reduction in the tenacity of the fibers, they also caused a notable increase in defects, including *neps*, thin places, and thick places. This ultimately led to a deterioration in the overall quality of the yarn. In particular, the tenacity of the fibers treated with anionic silica was found to decrease by 5.9% in comparison to the control, while the cationic silica treatment resulted in a 10.7% reduction in tenacity relative to the control. The reduction in strength, coupled with the rise in defects, underscores the detrimental impact of silica treatments on the mechanical properties and structural integrity of the yarn.

#### XRD

**Figure 5:**
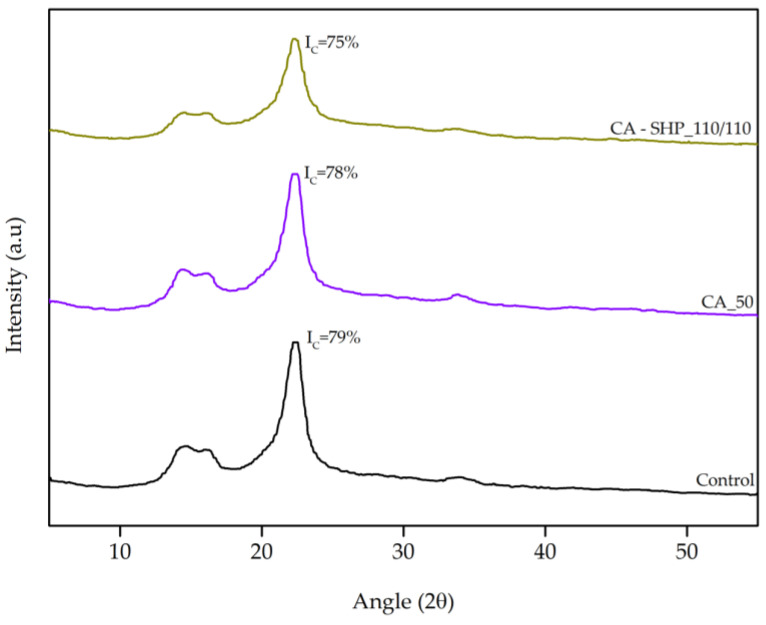
X-ray diffraction patterns of recycled cotton fibers untreated and after treatments.

An XRD analysis was conducted to study the crystallinity of recycled cotton fibers in samples that enhance the mechanical properties of the yarn, in addition to the corresponding control sample. The diffractograms are shown in Figure 5, as well as the calculated crystallinity index (I_C_). Figure 5 shows the XRD patterns of the recycled cotton fibers, both untreated and after undergoing CA treatment and CA-SHP_110/110 treatment. All three diffraction curves exhibit a crystalline structure characteristic of cellulose I [45]. The crystallinity index of the fibers was found to decrease following CA-SHP_110/110 treatment in comparison to both the CA treatment and the control sample. This reduction in crystallinity can be attributed to the elevated treatment temperature from 50 °C to 110 °C, which is further supported by the decrease in thermal stability observed in the same samples, as indicated by the shift of the cellulose DTG peak to lower temperatures (Figure 3b). The higher temperature likely disrupts the ordered crystalline regions, accelerate macromolecular degradation, leading to a less crystalline fiber structure [46]. The observed reduction in crystallinity for the CA-SHP_110/110-treated fibers may be linked to the partial disruption of the cellulose I crystalline structure due to elevated treatment temperatures. While this could lead to a reduction in strength, it may also increase flexibility and improve interfiber cohesion, contributing to improved yarn uniformity and fewer structural defects.

This dynamic equilibrium between crystalline order and processability may offer a partial explanation for the superior performance observed in mechanical testing. The reduction in the I_C_ due to the treatment temperature aligns with the improvement observed in the mechanical properties of the yarns produced from fibers treated with CA at 50 °C and CA-SHP at 110 °C. The decrease in I_C_ to 78% and further to 75% was accompanied by an increase in tenacity from 1.8% to 4.6%, as well as a reduction in thin places by 12% to 36% and in thick places by 6.1% to 10% (Table 5).

### 3.3. Knitted Fabric Characterization

#### 3.3.1. Air Permeability

**Figure 6:**
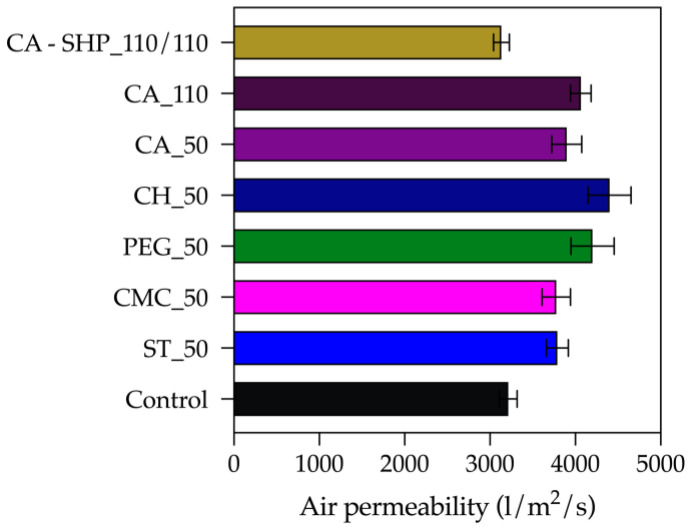
Air permeability of the knitted fabrics.

The air permeability values (l/m^2^/s) of the knitted fabric samples are presented in Figure 6. This study examines the air permeability of knitted fabrics containing 30% recycled cotton, focusing on the effects of various chemical treatments applied to the recycled fibers. Incorporating recycled cotton generally reduced air permeability compared to fabrics made entirely from virgin cotton. While shorter fibers might be expected to create a more open structure, higher twist levels and increased fiber entanglement instead result in a denser yarn and fabric, reducing porosity and restricting airflow.

The impact of chemical treatments on the air permeability of knitted fabrics was significant, with the majority of treatments resulting in increased permeability. However, CA-SHP_110/110 exhibited a different response, resulting in a reduction in air permeability. As demonstrated in Table 5, this treatment reduced *neps* by 7.0% compared to the control, yet its nep content remained higher compared to the samples CA_50, CH_50 and CA_110. Furthermore, the presence of thin and thick places was reduced by 36% and 10%, respectively, suggesting a more uniform and compact yarn structure. While a reduced number of *neps* is generally associated with increased permeability, the relatively elevated *neps* content in CA-SHP_110/110 in comparison to other samples may have resulted in increased fiber entanglement, leading to further compaction of the structure and restriction of airflow. A notable observation is that specific treated samples demonstrated higher air permeability compared to the control, irrespective of yarn quality. The phenomenon can be explained by the interaction between chemical treatments and the yarn and fabric structure. Lower-quality yarns, which are characterized by a higher occurrence of *neps* and thick and thin places, tend to create more open zones within the knitted fabric, allowing higher airflow. In contrast, higher-quality yarns, due to their increased uniformity and compactness, generally result in a denser fabric that restricts airflow. However, it has been demonstrated that specific chemical treatments can induce a relaxation of fibers and modify the yarn structure, thereby creating spaces that enhance permeability. The control sample, despite its intermediate structure, may exhibit a more regular but densely packed fiber arrangement, which would further restrict airflow. This underscores the notion that air permeability is not solely determined by the quality of the yarn, but also by the manner in which chemical treatments modify fiber structure and the subsequent interactions within the fabric. While the majority of treatments resulted in a loosening of the fabric structure and an enhancement of permeability, CA-SHP_110/110 demonstrated an effect of reinforcing fiber bonding, leading to the creation of a fabric that is both denser and less permeable.

#### 3.3.2. Thermal–Physiological Properties

An evaluation of the thermal properties of the knitted fabrics was conducted (Table 6), with measurements taken of thermal conductivity, diffusivity, absorptivity, resistance, flow, and thickness. The results indicate that chemical treatments significantly influence the thermal behavior of the fabrics. The thermal conductivity, which is indicative of the fabric’s capacity for heat transfer, exhibited a maximum value in CA-SHP_110/110 (48.5 W m^−1^ K^−1^), suggesting that its compact structure enhances heat conduction. Conversely, the lowest conductivity (43.4 W m^−1^ K^−1^) was observed in CH_50, a phenomenon that is attributable to its more porous structure, which reduces heat transfer. These results are consistent with those reported by Majumdar et al. (2010), who found that fabric porosity and yarn density are key determinants of thermal conductivity, with denser structures generally exhibiting higher values [47].

The thermal diffusivity, a key factor in determining the rate at which a fabric responds to temperature changes, was found to be lowest in ST_50 and CMC_50 (0.15 m^2^ s^−1^), suggesting that these fabrics possess a greater capacity to retain heat, which may offer enhanced insulation in cold conditions. Similarly, Majumdar et al. (2010) highlighted that fabrics with higher thickness and lower porosity exhibit superior heat retention properties [47]. The thermal absorptivity, which influences the initial warm or cool sensation upon contact with the skin, exhibited its highest value in ST_50 (118 s½ m^2^ K^−1^), thereby engendering a cooler feel, while PEG_50 demonstrated the lowest value (105 s½·m^2^·K^−1^), engendering a warmer sensation. The thermal resistance of the fabrics, indicative of their capacity to retain heat and provide insulation, exhibited a similar trend. PEG_50 exhibited the highest thermal resistance (21.0 m^2^·K·W^−1^), thereby indicating its potential for superior insulating properties. In contrast, ST_50 (19.2 m^2^·K·W^−1^), CMC_50, and CA_50 (19.3 m^2^·K·W^−1^) displayed lower thermal resistance, indicating reduced insulation capacity.

Thermal flow is defined as the amount of heat transferred through the fabric, with higher values indicating greater heat dissipation and a cooler sensation upon contact. CA_50 recorded the highest thermal flow, suggesting that these fabrics provide a noticeably cooling sensation. In contrast, PEG_50 exhibited the lowest value (0.69 W/m^2^), suggesting that it may induce a warmer sensation on the skin. The thickness of the knitted fabric also has a significant impact on thermal insulation. The thickest fabric, CA-SHP 110/110 (0.98 mm), exhibited higher thermal conductivity, facilitating heat transfer. Meanwhile, the thinnest fabric, designated CA_50 (0.85 mm), showed a reduced thermal resistance, a property that may partially account for its reduced insulating properties. Furthermore, Majumdar et al. [47] established a direct correlation between fabric thickness and insulation, proposing that the trapping of more air by thicker fabrics enhances thermal resistance.

#### 3.3.3. Pilling

As demonstrated in Table 7, an evaluation of the pilling resistance of the produced knitted fabrics was conducted, revealing that all the treatments applied to the recycled cotton fibers, with the exception of those treated with ST and CH, attained a rating of 4 or 4–5 in the pilling box trial. This outcome is directly related to yarn quality, particularly the presence of thick places, thin places, and *neps* [48]. A higher number of *neps* has been observed to correlate with increased pilling in knitted fabrics. However, for samples treated with CMC_50 and PEG_50, despite an increase in *neps*, no significant increase in pilling was observed, suggesting that the test had a reduced impact on these specific treatments, possibly due to their influence on fiber cohesion and surface characteristics. All the other treatments presented optimal resistance to pilling.

## 4. Conclusions

In this study, the mechanical properties of yarn produced from recycled cotton fibers were investigated considering ten different chemical treatments. The treatment combined with citric acid (CA) and sodium hypophosphite (SHP) significantly enhances the mechanical properties of recycled cotton fibers, particularly when the solution is preheated before fiber addition. The preheating process facilitates the formation of highly reactive cyclic anhydrides, which form robust ester bonds with cellulose, resulting in a 4.6% increase in fiber tenacity. Moreover, this treatment reduces yarn irregularities, with notable decreases in thin places (36%), thick places (10%), and *neps* (7%), leading to improved fiber quality and structural integrity. Additionally, no notable differences were observed in the ATR-FTIR spectra of the fibers treated with CA-SHP_110/110, suggesting that while ester bonds may form between citric acid and cellulose, the treatment does not significantly alter the overall chemical structure of the fibers. In conclusion, the combined CA-SHP_110/110 treatment provides an effective method for enhancing the mechanical performance and uniformity of recycled cotton fibers without significantly compromising their thermal stability or chemical structure. It is important to note that the results presented are specific to the 70:30 virgin-to-recycled cotton blend, selected based on its industrial viability and the optimal balance between spinnability and recycled content. The study did not include comparisons with 100% virgin or 100% recycled fiber yarns, which limits the generalizability of the findings. Further research is needed to assess whether similar improvements can be achieved at higher recycling ratios, and to determine the limits of processability and fabric quality under such conditions.

In addition to yarn-level improvements, fabric samples produced from the treated yarns were evaluated for thermal comfort, durability, and breathability through Alambeta testing, the pilling box test, and air permeability measurements, respectively. The collective outcomes of these evaluations support the conclusion that the treated fibers are suitably positioned for textile applications. While the current study focused on key mechanical and comfort-related properties, future work will include extended characterizations such as moisture absorption, wettability, flexural behavior, and flammability to comprehensively validate the performance of the fabrics for diverse end-use applications.

These developments demonstrate the potential for innovative treatments and processes to enhance the recycling and reuse of textile fibers, thereby facilitating more sustainable practices within the textile industry.

## Figures and Tables

**Table 1:** Methodology used for fiber modification by exhaustion.

N°	Sample	Conditions ^1^	Reagent	Concentration (%*w*/*v*)
1	PEG_50	50 °C, 1 h	PEG	1
2	CH_50		CH	0.05
3	CMC_50		CMC	0.2
4	ST_50		ST	1
5	CA_50		CA	1
6	CA_110	110 °C, 1 h	CA	1
7	CA-SHP_110		CA + SHP	1
8	CA-SHP_110/110	110 °C, 30 min 110 °C, 1 h	CA + SHP	1 1
9	SA_50	50 °C, 15 min pH 5–6	Anionic	1.5
10	SC_50		Cationic	1.5

^1^ 1.5 kg of the rinsed raw fiber and 15 L of the reagent solution were used for all the treatments. CMC—carboxymethylcellulose; PEG—polyethylene glycol; CA—citric acid; SHP—sodium hypophosphite; CH—chitosan; ST—starch; SA—anionic silica and SC—cationic silica.

**Table 2:** Pilling resistance rating according to ISO 12945-1:2020 [33].

Rating	Description	Point to be Taken into Consideration During the Assay
5	No change	No visual change
4	Slight change	Slight surface fuzzing
3	Moderate change	The test specimen may exhibit either or both of the following: (a) Moderate fuzzing; (b) Isolated fully formed pills
2	Significant change	Distinct fuzzing and/or pilling
1	Severe change	Dense fuzzing and/or pilling which covers the specimen

**Table 3:** Band assignments for FTIR-ATR spectra of the cotton recycled fibers.

Wavenumber (cm^−1^)	Allocations
3346–3267	O-H stretching vibration in cellulose II
2908	Asymmetric CH_2_ stretch: long alkyl chain
2848	Symmetric CH_2_ stretch: long alkyl chain
1717	C=O stretch
1640	Adsorbed H_2_O
1427	C-H wagging (in-plane bending)
1370	C-H bending (deformation stretch)
1314	C-H wagging
1205	OH in-plane bending
1160–1110	Asymmetric stretching of the C-O-C of the β-glycosidic bond
1030	C-O stretch
890	C-O-C in plane, vibration due to symmetric stretching of the β-glycosidic bond

**Table 4:** Thermogravimetric analysis parameters of recycled cotton fibers untreated and after treatments.

	T_max_ (°C)	Weight Loss (%)	Residue (%)
Control	368	77	12.4
ST_50	371	81	9.07
CMC_50	371	83	6.19
PEG_50	368	82	7.89
CH_50	371	83	6.02
CA_50	356	87	5.63
CA_110	356	81	8.02
CA-SHP_110	332	62	22.2
CA-SHP_110/110	352	77	11.8
SA_50	368	81	8.26
SC_50	352	81	9.68

**Table 5:** Mechanical tests and quality assessment of yarns with recycled cotton fibers, highlighting the percentage improvement of each treatment when compared to the control sample.

	Tenacity (cN/tex)		Thin Places (50%/km)		Thick Places (50%/km)		*Neps* (200%/km)	
Control	14.63 ± 0.22		176 ± 5		2013 ± 60		2269 ± 68	
ST_50	15.34 ± 0.23	+4.9%	309 ± 55	+76%	2361 ± 104	+17%	2746 ± 173	+21%
CA-SHP_110/110	15.31 ± 0.23	+4.6%	113 ± 28	−36%	1813 ± 107	−10%	2119 ± 132	−7.0%
CMC_50	15.23 ± 0.23	+4.1%	246 ± 54	+40%	2202 ± 106	+9.4%	2571 ± 106	+13%
PEG_50	14.91 ± 0.22	+1.9%	242 ± 50	+38%	2278 ± 38	+13%	2828 ± 84	+25%
CA_50	14.89 ± 0.22	+1.8%	155 ± 42	−12%	1890 ± 111	−6.1%	2066 ± 82	−8.9%
CH_50	13.99 ± 0.21	−4.3%	231 ± 49	+31%	1944 ± 129	−3.4%	1963 ± 114	−13%
CA_110	13.69 ± 0.21	−6.4%	110 ± 51	−38%	1610 ± 203	−20%	1641 ± 232	−28%
SA_50	13.76 ± 0.21	−5.9%	1829 ± 431	+939%	3891 ± 252	+93%	4163 ± 322	+83%
CA-SHP_110	13.51 ± 0.20	−7.7%	263 ± 56	+49%	2297 ± 55	+14%	2382 ± 114	+5.0%
SC_50	13.06 ± 0.20	−10.7%	3131 ± 819	+1679%	4807 ± 368	+139%	6248 ± 449	+175%

**Table 6:** Thermal–physiological properties from the knitted fabric.

	Thermal Conductivity l (W m^−1^ K^−1^)	Thermal Diffusivity a (m^−2^s^−1^)	Thermal Absorptivity b (s^1/2^ m^−2^ K^−1^)	Thermal Resistance R (m^2^ K W^−1^)	Thermal Flow q_máx_ (W m^−2^)	Thickness (mm)
Control	46.9 ± 0.57	0.18 ± 0.00	110 ± 2.16	20.1 ± 0.26	0.73 ± 0.07	0.94 ± 0.00
ST_50	45.6 ± 0.69	0.15 ± 0.01	118 ± 0.82	19.2 ± 0.12	0.76 ± 0.03	0.88 ± 0.01
CMC_50	45.8 ± 0.21	0.15 ± 0.01	117 ± 2.36	19.3 ± 0.00	0.75 ± 0.06	0.87 ± 0.01
PEG_50	44.3 ± 1.06	0.17 ± 0.01	105 ± 2.05	21.0 ± 0.29	0.69 ± 0.03	0.93 ± 0.02
CH_50	43.4 ± 0.43	0.16 ± 0.00	109 ± 2.49	20.6 ± 0.12	0.75 ± 0.07	0.88 ± 0.02
CA_50	44.5 ± 1.00	0.17 ± 0.01	109 ± 1.41	19.3 ± 0.45	0.77 ± 0.04	0.85 ± 0.01
CA_110	44.2 ± 0.87	0.17 ± 0.01	108 ± 2.36	19.5 ± 0.74	0.74 ± 0.02	0.86 ± 0.04
CA-SHP__110/110	48.5 ± 0.50	0.17 ± 0.00	115 ± 2.16	20.5 ± 0.75	0.75 ± 0.02	0.98 ± 0.00

**Table 7:** Pilling measurements from the knitted fabric.

	Trial	
	1	2
Control	5	5
ST_50	4	4
CMC_50	5	5
PEG_50	5	5
CH_50	4–5	4–5
CA_50	5	5
CA_110	5	5
CA-SHP__110/110	5	5

## Data Availability

The original contributions presented in this study are included in the article. Further inquiries can be directed to the corresponding author.
